# Cytokinins and Expression of *SWEET, SUT, CWINV* and *AAP* Genes Increase as Pea Seeds Germinate

**DOI:** 10.3390/ijms17122013

**Published:** 2016-12-01

**Authors:** Paula E. Jameson, Pragatheswari Dhandapani, Ondrej Novak, Jiancheng Song

**Affiliations:** 1 School of Biological Sciences, University of Canterbury, Private Bag 4800, Christchurch 8140, New Zealand; prapthis@yahoo.co.in (P.D.); jcsong88@yahoo.com (J.S.); 2 Laboratory of Growth Regulators, Centre of the Region Haná for Biotechnological and Agricultural Research, Institute of Experimental Botany CAS & Faculty of Science of Palacký University, Šlechtitelů 27, 783 71 Olomouc, Czech Republic; ondrej.novak@upol.cz; 3 School of Life Sciences, Yantai University, Yantai 264005, China

**Keywords:** cytokinin, germination, *Pisum sativum*

## Abstract

Transporter genes and cytokinins are key targets for crop improvement. These genes are active during the development of the seed and its establishment as a strong sink. However, during germination, the seed transitions to being a source for the developing root and shoot. To determine if the sucrose transporter (*SUT*), amino acid permease (*AAP*), Sugar Will Eventually be Exported Transporter (*SWEET*), cell wall invertase (*CWINV*), cytokinin biosynthesis (*IPT*), activation (*LOG*) and degradation (*CKX*) gene family members are involved in both the sink and source activities of seeds, we used RT-qPCR to determine the expression of multiple gene family members, and LC-MS/MS to ascertain endogenous cytokinin levels in germinating *Pisum sativum* L. We show that genes that are actively expressed when the seed is a strong sink during its development, are also expressed when the seed is in the reverse role of being an active source during germination and early seedling growth. Cytokinins were detected in the imbibing seeds and were actively biosynthesised during germination. We conclude that, when the above gene family members are targeted for seed yield improvement, a downstream effect on subsequent seed germination or seedling vigour must be taken into consideration.

## 1. Introduction

The dynamic relationship between sources and sinks changes markedly during the life cycle of the plant. Leaves commence their life cycle initially as sinks, and mature into sources, and the seed, initially a strong sink, becomes the source of energy and nutrients during germination [1]. The availability and partitioning of carbon and nitrogen (N) provide the resources underpinning source-sink dynamics [2,3].

Key to the movement of sucrose around the plant are the Sugar Will Eventually be Exported Transporters (SWEETs), sucrose transporters (SUTs) and cell wall invertases (CWINVs) [1,4,5], and to the movement of amino acids are the amino acid permeases (AAPs) [6,7]. These same proteins are involved in the transport of assimilates to the developing seed [8,9,10,11,12]. However, during germination, as the seed changes from a sink to a source, the question arises as to whether these proteins are involved in the re-mobilisation of the reserves they helped to mobilise to the seed in the first place. 

Functional studies have shown that the *Arabidopsis* AAPs can transport a wide range of amino acids [6,13], and various gene family members are involved in the source to sink translocation of amino acids [7]. In pea, expression of the *AtAAP1::PsAAP1* construct increased overall plant biomass and N content, as well as increasing seed yield and seed N [11]. Seed storage proteins are degraded to provide amino acids for the biosynthesis of nucleic acids and new proteins, but may also be metabolised to satisfy the energy demands of the developing seedling [14,15]. Transportation of amino acids within the germinating seed and to the young seedling would appear to be a necessary requirement. In both the legume, *Medicago truncatula*, and in rice, the *AAP*s constitute larger gene families than in *Arabidopsis* [13,16]. We showed previously that of the 13 *PsAAP* gene family members detected in a pea transcriptome, the *PsAAP*s were represented in most of the clusters of *AAP* orthologues in *Arabidopsis* and other leguminous species [17]. Multiple sequences were identified in several family members, particularly for *PsAAP2* (Cluster 3A) and *PsAAP7* (Cluster 1), as previously noted [13]. *AtAAP7* has yet to be functionally characterised. 

In contrast to the *AAP*s, *SUT*s belong to a smaller gene family [18]. SUTs are considered essential to the movement of sucrose from source leaves to sink organs, and to the uploading of sucrose into seeds [9,19,20]. Type I and II SUTs are localised to the plasma membrane [20]. The role of SUTs during early seed germination has been studied in rice [21,22]. In monocots, Type II SUTs (OsSUT1, 3, and 4) are utilised for phloem loading [20,23]. During early seed germination, *OsSUT1* is upregulated in the scutellar vascular bundle [21], while *OsSUT4* is expressed in the seed aleurone layer, and subsequently in the scutellum and embryonic vascular bundle [22], indicating differential temporal and spatial expression for these gene family members during germination. 

In *Ricinus communis*, sucrose is released from the endosperm to the apoplast and from there taken up by the cotyledons for transfer to the rest of the seedling [24]. *RcSUT1* was shown to be expressed more in the cotyledon of the germinating seed than in the endosperm [24]. While the loading of sucrose into developing legume seeds has been studied in detail [8,9,19,25,26], it would appear less attention has been given to the movement of sugars during the germination of non-endospermous seed, such as pea.

*SWEETs* are the most recent gene family to be designated a key role in sink/source dynamics [27,28]. Dhandapani et al. (2016) [17] showed that the 13 *PsSWEET* gene sequences identified from a pea transcriptome had members in all four of the *SWEET* clades described recently [12,29]. *SWEET*s have been strongly linked to development in reproductive tissues, especially seeds [12,30,31]. Transgenic analysis has indicated that *SWEET*s may be activated during seed germination. Seeds over-expressing *AtSWEET16* (a Clade IV *SWEET*) germinated faster than controls [31] as did those over-expressing *AtSWEET4* (a Clade II *SWEET*) [32]. However, the expression of endogenous *SWEET*s during germination has yet to be shown. 

Cell wall invertases catalyse the irreversible breakdown of sucrose to fructose and glucose, and are an integral component of the movement of sucrose between sources and sinks [1,33,34]. They have been shown to be up-regulated in several scenarios affecting source-sink relationships, such as the cytokinin-induced delay of senescence [35,36]. However, they appear to have a dual role, being involved also in stimulating the cell cycle through the production of sugar signals, the latter implicating them, along with the cytokinins, in cell division and seed development [37,38,39,40]. Cell wall invertases are likely also to be involved in the mobilisation of resources during seed germination.

The cytokinins are clearly implicated in seed development [41]. The cytokinins are biosynthesised by isopentenyl transferase (IPT), degraded by cytokinin oxidase/dehydrogenase (CKX), and conjugated to storage or inactivated forms by glycosidases. The first formed cytokinins are nucleotides, which may be activated to the free base forms by LONELY GUY (LOG) [42]. A signal transduction pathway is activated upon the detection of free base cytokinins by receptors [43], which activate response regulators (RR) downstream (for recent reviews, see [44,45]). 

The feeding of radioactively-labelled precursors has shown that germinating lupin and maize seeds are capable of biosynthesising cytokinin, but that this is restricted to the embryo axis [46,47,48,49]. In maize, the cytokinin then moves unidirectionally from the embryo to accumulate in the endosperm in maize [48]. The cytokinins synthesized by the embryonic axis of lupin, and which are also transported unidirectionally to the cotyledons [46], were shown to be highly stable and to induce cotyledon expansion and chlorophyll synthesis [47]. Further, the regulation of reserve mobilization in yellow lupin seeds and in germinating chick-peas, appears to be mediated, at least in part, by cytokinin emanating from the embryonic axis [49,50]. Differential activity of different cytokinin forms has also been suggested in germinating chick-pea with zeatin riboside (ZR) affecting the mobilisation of carbohydrate, whereas zeatin (Z) had more impact on the protein in the cotyledons; isopentenyl adenine (iP) affected only the metabolism of carbohydrates, whereas iPR (iP riboside) mainly affected lipid metabolism [50,51]. Interestingly, all enzymes of the isoprenoid pathway have increased in activity within 2 to 6 h from the start of imbibition [52], providing precursors to the cytokinin biosynthetic machinery.

As transporter genes (reviewed in [10,53]), and cytokinin biosynthetic and degradative genes (reviewed in [41]) are the targets of transgenic approaches to increasing seed quality and/or quantity, we were interested if these gene families were expressed during germination. Such knowledge is important as changes in the expression of genes in the parental generation may impact seed germination and seedling vigour of the subsequent generation. 

We chose to work with pea as it is both an important legume crop and a well-studied model crop. Additionally, pea seeds are non-dormant and non-endospermous, and their covering layers are not a mechanical constraint to radical protrusion [54]. The germination process is divided into an initial rapid imbibition phase during which pea seeds exhibit increased respiration and metabolic activity [55], followed by an activation phase during which stored carbohydrate and protein are mobilised [56]. Our aim was to determine if the gene families known to be involved in sink activity during seed development (*SWEET*s, *SUT*s, *CWINV*s, *IPT*, *LOG*, *CKX*) are also involved in mobilising reserves during germination. Based on gene family members detected in our pea transcriptome [17], we used RT-qPCR to monitor their expression. We show that genes actively expressed when the seed is a strong sink during its development are also actively expressed when the seed is in the reverse role of being an active source during germination.

## 2. Results

**Figure 1:**
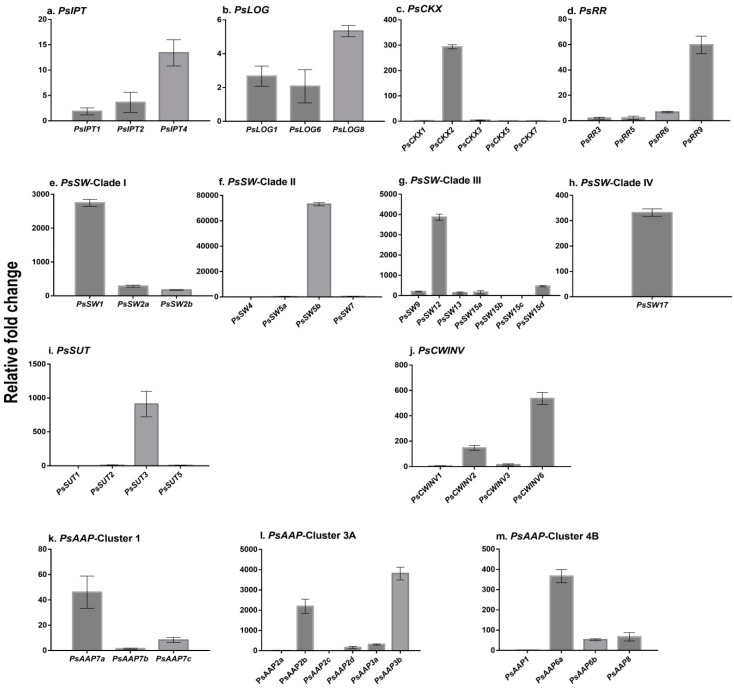
Relative expression of cytokinin biosynthesis (*PsIPT*), activation (*PsLOG*), degradation (*PsCKX*) and response regulator (*PsRR*) gene family members along with *PsSWEET* (*PsSW*), *PsSUT*, *PsCWINV* and *PsAAP* gene family members in *Pisum sativum* cotyledons after four hours of imbibition. Fold-change values were calculated using *PsEF*, *U18S*, *PsGAP* and *PsACT* as internal controls using three technical replicates for each of two biological replicates in the RT-qPCR. The results are expressed as ± SD.

**Figure 2:**
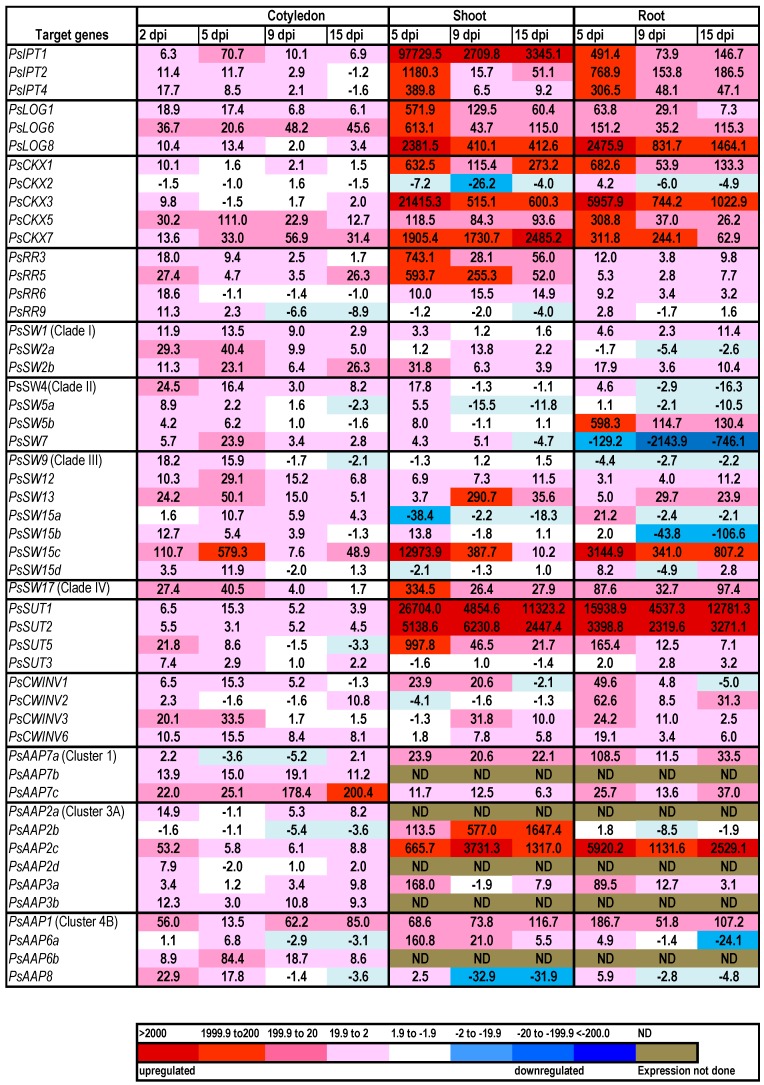
Relative expression of cytokinin biosynthesis (*PsIPT*), activation (*PsLOG*) degradation (*PsCKX*) and response regulator (*PsRR*) gene family members along with *PsSWEET*, *PsSUT*, *PsCWINV* and *PsAAP* gene family members in *Pisum sativum* cotyledons at two days post-imbibition (dpi), and in cotyledons, shoots and roots at 5, 9 and 15 dpi. Values are fold-changes relative to the expression at four hours post-imbibition (4 hpi). The colour scale indicates up-regulated expression (red scale), similar (white) and down-regulated expression (blue scale) relative to 4 hpi.

Expression relative to the reference genes is shown for all gene family members at four hours post-imbibition (4 hpi) (Figure 1). This timing coincides with Phase I water uptake during imbibition by peas and is prior to mass reserve mobilisation [52]. The data for subsequent time points are shown in a heat map as fold-change relative to 4 hpi (Figure 2). 

### 2.1. Cytokinin Biosynthesis and Metabolism in the Germinating Pea 

The three *PsIPT* gene family members identified from the transcriptome were expressed in cotyledons at 4 hpi (Figure 1). Expression of the three *PsIPT* gene family members increased in cotyledons as they germinated, but reduced at later stages. Expression of *PsIPT* was at its greatest in the emerging roots and shoots (Figure 2). The three *LOG* family members expressed in cotyledons, roots and shoots. Expression in the cotyledons at 4 hpi was low (Figure 1) relative to later stages of germination (Figure 2). Relative to 4 hpi, the *PsLOGs* were expressed in the developing shoots and the elongating roots, particularly *PsLOG8* (Figure 2). 

Relative to the other three *PsCKX* family members, *PsCKX2* was more strongly expressed within 4 hpi (Figure 1). This level changed little in the germinating cotyledon (hence, showing as “no change” on the heat map (Figure 2)). By 2 dpi, *PsCKX5* and *7* had increased in the cotyledon. *PsCKX2* remained constitutively expressed as roots and shoots developed, whereas *PsCKX1*, *3*, *5* and *7* increased substantially in the developing shoots and roots (Figure 2). Of the four response regulators, which are putative Type As [17], *PsRR9* was expressed within 4 hpi. All family members showed increased expression in cotyledons at 2 dpi relative to 4 hpi. *PsRR3* and *5* were strongly expressed in the developing shoot, but much less strongly expressed in the elongating root (Figure 2). In imbibing seeds at 4 hpi, only cytokinin free bases and ribosides were detected, including both *trans* zeatin (*t*Z) and isopentenyladenine (iP) [17], but much lesser levels of dihydrozeatin (DZ) and *c*Z (Table 1). No *O*- or *N*-glucosides (Table 1) nor nucleotides ([17]; Table 1) were detected at 4 hpi. Two days post-imbibition, the cytokinin levels had increased substantially, to some extent due to an increase in *t*Z, but mostly due to the more substantial increase in nucleotides, particularly iPRMP (iP ribosyl monophosphate) [17] and, to a lesser extent, *cis* zeatin riboside monophosphate (cZRMP) and tZRMP. Cytokinin levels in the cotyledons peaked at 11 days and then declined, due to a decrease in nucleotides ([17]; Table 1). Developing shoots, first analysed five days after imbibition, had the greatest amount of cytokinin relative to later stages and predominantly as nucleotides with iPRMP ≅ cZRMP >>tZRMP (Table 1). Emerging roots at five days post-imbibition, had less cytokinin then shoots, but more than cotyledons on a DW basis. In contrast to shoots, the cytokinin level in roots increased to a peak at 11 dpi, and then declined. Again the major contributors to the cytokinin content were the nucleotides with iPRMP ≅ cZRMP > tZRMP, with free bases and ribosides contributing to a lesser extent (Table 1). Cytokinin *O*-glucosides were not detected at 4 hpi, but accumulated over time in cotyledons, shoots and roots. Zeatin 9-glucosides accumulated to low levels in seedling roots and shoots (Table 1), but no 7-glucosides were detected. 

### 2.2. Expression of Transporter Genes in the Germinating Seed

The relative expression of the *PsSWEET* gene family members differed in the germinating seeds within 4 hpi with, for example, extremely low values (<5) for Clade III *PsSW15b* and *15c* and extremely high values (ca. 70,000) for Clade II *PsSW5b* (Figure 1). Generally, the *PsSWEET*s increased in expression in the cotyledons following imbibition. *PsSWEET1*, *5b* and *12* were generally constitutively expressed in the developing shoots and roots. *PsSWEET15c* was strongly expressed in the younger developing shoots and roots. Clade IV *PsSWEET17* was elevated in expression in cotyledons, shoots and roots (Figure 2). 

Type II *PsSUT3* was elevated relative to the three Type I *SUTs* in the imbibing seeds at 4 hpi (Figure 1), and was subsequently more-or-less constitutively expressed (Figure 2). In the cotyledons, there was generally an increase in the expression of the other *PsSUTs* as the seeds germinated (Figure 2). However, relative to the expression at 4 hpi, both *PsSUT1* and *2* were very strongly expressed in both developing shoots and roots. 

The four *PsCWINV* gene family members were expressed in the imbibing seeds, particularly *PsCWINV6* (Figure 1), with expression generally increasing in the cotyledons with germination. Expression was more consistently elevated in roots than in shoots (Figure 2). 

Most of the 13 *PsAAP* gene family members were expressing in cotyledons within 4 hpi, with two Cluster 3A gene family members strongly expressed (Figure 1). There was an increase in expression of the 13 *PsAAP* gene family members in germinating seeds, generally peaking at 2 dpi or 5 dpi in the cotyledons (Figure 2). Strong expression of *PsAAP2c* was evident in both roots and shoots, and of *2b* in shoots. Lower level but consistent expression was apparent for *PsAAP3a* (Cluster 3A) and *PsAAP1* and *6a* (Cluster 4B) across organs. *PsAAP7* expressed increasingly strongly over time, whereas *PsAAP8* showed low and decreasing expression over time.

## 3. Discussion 

Germinating seeds are metabolically highly active, exhibiting increased respiration and metabolic activity within a few hours of imbibition, followed by the degradation of carbohydrate, lipid and protein stores, and the mobilisation of these to the embryo [57,58]. Pea stores both carbohydrate and protein that must be metabolised and transported from the cotyledons to the embryo axis and thence to the elongating root and shoot. Cytokinins and expression of *SWEET*s*, SUT*s, *CWINV*s and *AAP*s were all detected within four hours of commencement of imbibition. Within two days of imbibition, at which stage the radicle and plumule had emerged, increased expression of most gene families was occurring. 

Within 4 hpi, expression of *PsIPT* was detected as were low levels of biologically active cytokinins. As the seeds germinated, the accumulation of cytokinin nucleotides was strong evidence of cytokinin biosynthesis occurring [59], and the elevated expression of response regulators indicated that the cytokinin signal transduction pathway was operational [36]. This strongly supports the contention that cytokinin emanating from the embryonic axis of legumes is biosynthesised in situ and is involved in early reserve mobilisation [49,51]. As roots and shoots emerged, increased cytokinin biosynthesis was apparent. The increase in nucleotides as the seeds germinated is similar to that reported for germinating *Tagetes minuta* L. [60]. Earlier work with chick-peas and more recent work with pea did not report nucleotide levels [50,56] but these appear to be the most significant cytokinin form in the germinating seed and during early seedling growth ([17]; Table 1). The origin of the cZRMP is of interest. It has yet to be determined whether the cytokinin released from tRNA (the usually cited source of the *cis* cytokinins) has been phosphorylated, making it then accessible to LOG, or whether it has been directly biosynthesised.

The active forms of the cytokinins are the free bases [43] which are present at low levels in the imbibing seed [17] and are particularly evident in the emerging shoot and root (Table 1). Several of the earlier papers [46,50,60] refer to the dihydro-derivatives, which are not metabolised by CKX. DHZRMP, DHZ and DHZR were detected in pea but only at low levels in the cotyledons and in the emerging shoots and roots. Cytokinin biosynthesis (as determined by expression of *PsIPT* and the levels of nucleotides) was more strongly elevated in early shoot growth compared with the roots. Most notable though were the elevated levels of the RR genes in shoots compared with roots, indicating a stronger response to the cytokinin in the shoots compared with the roots. This aligns with our knowledge that cytokinin is known to promote shoot growth and inhibit aspects of root growth [61].

It is important to note that *CKX* expression was also increasing during germination, and was strongly upregulated in the young seedling shoots and roots. An increase in *CKX* at the time when cytokinin levels are increasing is a common phenomenon, and is indicative of homeostatic mechanisms operating [41,45]. *CKX* activity is the likely reason for the levels of free bases and ribosides being significantly less than those of the nucleotides: strong biosynthesis and metabolism of cytokinin are occurring during early seedling growth. 

As decreasing *CKX* activity is a target for enhancing yield in both monocots and dicots [41,62,63] it is important to be aware that any increased cytokinin may impact source-sink relationships. Particularly critical in peas may be *PsCKX2,* which is expressed in the imbibing seed: if this gene family member were to be down-regulated, this may impact severely on the release of nutrients from the cotyledon. Down-regulation of *PsCKX7* may have a double impact on the root by stimulating competitive sink activity in the shoot, as well as inhibiting root growth through elevated endogenous cytokinin [61].

Transgenic work has implicated both a Clade II and a Clade IV *SWEET* in the germination of *Arabidopsis* seeds [31,32]. We show here that several *PsSWEET* gene family members are strongly expressed in germinating pea seeds with *PsSWEET5b* (a Clade II *SWEET*) very strongly expressed within 4 hpi and PsSW17 (a Clade IV *SWEET*) strongly up-regulated during germination. 

Elevated expression of *PsCWINV* gene family members occurred during germination and early seedling growth, again supporting a strong link between both cytokinin and CWINVs [35,38,39,40], and INV and SWEETs [12], with the likelihood of CWINVs converting sucrose to hexoses, available to Clade II SWEETs. Subsequently, Clade III SWEETs were also activated to move sucrose towards SUTs for uploading into the phloem. *PsSUT3* appears to be more-or-less constitutively expressed, but both *PsSUT1* and *2* are very strongly unregulated in the elongating root and shoot, particularly compared to their activity in the cotyledons. 

The pea homologues of the *AtAAP* gene family members linked to seed loading in *Arabidopsis* (Cluster 4B, *AtAAP1* and *8*) [64,65] were not particularly strongly expressed in the imbibing pea seed. However, *PsAAP1* was strongly upregulated in the cotyledons, shoots and roots as the seed germinated. Recently, Santiago and Tegeder (2016) suggested that *AtAAP8* was the “long sought after phloem loader”, showing it capable of loading a broad spectrum of amino acids into the phloem [7]. Interestingly, this gene family member was not strongly expressed during germination, relative to some other *AtAAP* gene family members, and was barely expressed during early pod and seed development in pea (our unpublished data). Indeed, *PsAAP8* was significantly down-regulated in the elongating shoots, in contrast to Cluster 3A member, *PsAAP2b*, which was specifically up-regulated in the shoot compared to the roots. *PsAAP2c* was the most highly expressed *AAP* gene family member in both the elongating roots and shoots, indicating clear differential expression of the *PsAAP* gene family members.

While targeting of nutrient transporters has led to increased yield in some instances [53], the impact of that manipulation has not been reported on the germinating seed. In this work we show that key gene families involved in seed development, some of which have been the targets of genetic manipulation, are also involved in germination. This information is important, as potential imbalances in the mobilisation of carbon and nitrogen resources may have detrimental effects on the developing seedling, as shown for *Arabidopsis* overexpressing *SWEET16* under nitrogen-limiting conditions [31]. 

## 4. Materials and Methods

### 4.1. Plant Material and Sample Preparation

Surface sterilised seeds of *Pisum sativum* variety Bohatyr were imbibed for 4 h with stirring in Klambt medium [66] and placed in sterilised 500 mL containers with 0.6% (*w*/*v*) agar and 10% (*w*/*v*) Hoagland’s mineral salts solution [67]. The containers were placed in a growth room at 22 °C with a 16-h photoperiod. Five imbibed or germinating seeds/seedlings were sampled at 4 h, 2 days, 5 days, 9 days and 15 days after imbibition, submerged briefly in liquid nitrogen and stored at −80 °C until use.

### 4.2. RNA Isolation and Target Gene Isolation

Total RNA was extracted from each sample using TRIzol Reagent (Invitrogen, Carlsbad, CA, USA) and converted to cDNA as previously described [17]. Sequences of candidate gene family members were isolated from an RNA-seq transcriptomic data set as described in [17]. Primers were designed for the RT-qPCR and their products sequenced. The primers used are reported in [17]. 

### 4.3. Real-Time Reverse Transcription Quantitative PCR (RT-qPCR)

The relative expression levels of each of the genes of interest were determined using RT-qPCR as described in [68], with two biological replicates and three technical replicates for each sample. PCR was performed in a Rotor-Gene Q (Qiagen, Hilden, Germany), with either a home-made SYBR Green master mix or the KAPA SYBR^®^ FAST qPCR Kits (Kapa Biosystems, Boston, MA, USA). Four reference genes, *PsEF*, *PsGAP*, *PsACT* and *U18S* were used to correct the *C*_t_ values before calculating the relative expression of each sample as described in [68]. 

### 4.4. Cytokinin Analyses

Cotyledons from four individual imbibed seeds or seedlings, making four biological replicates, were ground under liquid nitrogen and freeze-dried. Cytokinins were extracted and purified as described in [17,69] and subjected to analysis by an LC-MS/MS system consisting of an ACQUITY UPLC^®^ System (Waters, Milford, MA, USA) and Xevo^®^ TQ-S (Waters) triple quadrupole mass spectrometer. Quantification was obtained using multiple reaction-monitoring (MRM) mode of selected precursor ions along with stable isotope internal standards [69].

## Figures and Tables

**Table 1:** Endogenous cytokinins in germinating pea seeds. The data are the averages of four biological replicates and are expressed as ± SD.

Cytokinin Levels	Cotyledon						Shoot				Root			
(pmol/g DW)	4 hpi	2 dpi	5 dpi	11 dpi	15 dpi	25 dpi	5 dpi	11 dpi	15 dpi	25 dpi	5 dpi	11 dpi	15 dpi	25 dpi
**Total cytokinin**	5.05 ± 0.4	38.5 ± 7.3	51.37 ± 4.4	77.18 ± 11.4	47.23 ± 2.2	28.65 ± 2.2	357.41 ± 17.3	93.97 ± 14.9	55.83 ± 2.4	43.68 ± 1.3	197.89 ± 14.4	220.5 ± 26.8	113.68 ± 9.1	94.11 ± 5.9
**Total nucleotides**	<lod	34.5 ± 1.9	43.13 ± 3.8	64.73 ± 10.5	20.29 ± 2.8	9.39 ± 1.8	289.8 ± 13.6	65.44 ± 11.3	30.05 ± 2.8	17.64 ± 0.9	134.03 ± 13.3	162.86 ± 24.3	47.96 ± 5.0	31.61 ± 5.8
**Total bases**	3.72 ± 0.3	7.27 ± 0.6	3.72 ± 0.3	5.55 ± 0.5	2.75 ± 0.9	4.11 ± 0.3	17.31 ± 1.5	7.29 ± 1.1	5.9 ± 1.5	6.31 ± 0.5	12.65 ± 1.2	10.62 ± 0.3	8.97 ± 1.6	10.39 ± 0.5
**Total ribosides**	1.33 ± 0.1	3.56 ± 0.4	2.57 ± 0.4	3.07 ± 0.3	3.94 ± 0.4	2.36 ± 0.4	32.38 ± 3.3	8.36 ± 1.6	7.16 ± 0.5	3.71 ± 0.5	33.42 ± 2.0	26.24 ± 1.9	37 ± 8.2	13.87 ± 2.5
**Total *O*-glucosides**	<lod	2.08 ± 0.3	1.95 ± 0.2	3.75 ± 0.9	19.68 ± 0.9	11.81 ± 2.2	17.92 ± 1.5	<lod	12.49 ± 1.3	15.04 ± 1.8	17.48 ± 1.6	19.58 ± 1.3	19.03 ± 2.9	34.56 ± 4.2
**Total *N*-glucosides**	<lod	<lod	<lod	0.07 ± 0.02	0.64 ± 0.2	1.13 ± 0.4	<lod	<lod	0.24 ± 0.1	0.97 ± 0.1	0.34 ± 0.02	1.2 ± 0.3	0.73 ± 0.1	3.68 ± 0.4
**tZRMP**	-	-	-	-	-	-	23.87 ± 2.1	11.41 ± 1.8	2.19 ± 0.3	0.61 ± 0.04	15.17 ± 1.9	37.66 ± 6.9	6.68 ± 1.8	1.27 ± 0.4
**DHZRMP**	<lod	<lod	<lod	0.1 ± 0.01	<lod	0.27 ± 0.1	3.18 ± 0.1	0.66 ± 0.1	1.05 ± 0.3	0.89 ± 0.1	1.61 ± 0.3	0.83 ± 0.2	0.58 ± 0.1	0.26 ± 0.1
**iPRMP**	-	-	-	-	-	-	142.08 ± 9.9	22.66 ± 4.5	10.76 ± 1.3	6.91 ± 0.5	69 ± 4.02	68.53 ± 15.6	16.32 ± 1.0	10.09 ± 3.0
**cZRMP**	<lod	10.4 ± 1.2	17.25 ± 2.7	43 ± 9.1	15.07 ± 1.8	6.34 ± 1.1	120.66 ± 3.9	30.98 ± 6.6	17.76 ± 0.8	9.23 ± 0.5	48.25 ± 10.8	55.83 ± 9.0	24.46 ± 3.03	19.99 ± 3.1
**tZ**	-	-	-	-	-	-	7.16 ± 0.6	3.81 ± 0.8	1.74 ± 0.1	1.74 ± 0.1	4.55 ± 0.5	5.75 ± 0.5	3.83 ± 1.0	1.33 ± 0.1
**DHZ**	0.24 ± 0.04	0.1 ± 0.02	0.04 ± 0.01	<lod	0.09 ± 0.01	0.22 ± 0.1	0.49 ± 0.1	<lod	0.77 ± 0.1	1.26 ± 0.1	0.29 ± 0.1	<lod	0.41 ± 0.1	0.38 ± 0.02
**iP**	-	-	-	-	-	-	7.34 ± 0.7	2.79 ± 1.0	2.63 ± 1.0	2.41 ± 0.3	5.82 ± 0.8	3.62 ± 0.2	3.53 ± 0.6	3.22 ± 0.4
**cZ**	0.16 ± 0.03	0.15 ± 0.03	0.19 ± 0.02	1.01 ± 0.2	0.62 ± 0.1	0.68 ± 0.1	2.31 ± 0.3	0.69 ± 0.1	0.76 ± 0.1	0.91 ± 0.1	2.1 ± 0.2	1.25 ± 0.2	1.2 ± 0.1	5.46 ± 0.9
**tZR**	-	-	-	-	-	-	4.01 ± 0.3	1.86 ± 0.3	0.47 ± 0.1	0.28 ± 0.1	6.78 ± 0.4	9.13 ± 0.7	6.21 ± 1.4	0.75 ± 0.2
**DHZR**	0.17 ± 0.1	0.39 ± 0.1	0.15 ± 0.02	0.2 ± 0.03	0.12 ± 0.0	0.18 ± 0.04	2.85 ± 0.3	1.65 ± 0.4	0.69 ± 0.1	0.63 ± 0.1	2.18 ± 0.1	2.28 ± 0.2	0.85 ± 0.2	0.8 ± 0.2
**iPR**	-	-	-	-	-	-	13.12 ± 2.5	2.88 ± 0.8	2.98 ± 0.8	1.33 ± 0.1	19.19 ± 1.7	11.24 ± 1.9	24.44 ± 5.8	7.81 ± 2.0
**cZR**	0.89 ± 0.1	0.65 ± 0.03	0.77 ± 0.1	1.57 ± 0.2	2.66 ± 0.5	1.19 ± 0.3	12.41 ± 2.3	1.97 ± 0.3	3.02 ± 0.8	1.47 ± 0.3	5.27 ± 0.1	3.59 ± 0.5	5.5 ± 1.0	4.51 ± 0.3
**tZOG**	-	-	-	-	-	-	1.42 ± 0.14	4.03 ± 0.8	1.74 ± 0.14	1.5 ± 0.1	0.73 ± 0.1	4.04 ± 0.6	1.94 ± 0.2	0.9 ± 0.1
**DHZOG**	<lod	<lod	0.03 ± 0.0	0.15 ± 0.02	0.3 ± 0.03	0.31 ± 0.04	0.32 ± 0.04	1.25 ± 0.2	2.71 ± 0.3	6.79 ± 1.1	0.19 ± 0.01	1.41 ± 0.1	1.05 ± 0.1	0.34 ± 0.03
**cZOG**	<lod	1.26 ± 0.1	0.98 ± 0.1	1.76 ± 0.4	9.63 ± 0.3	4.45 ± 0.8	2.54 ± 0.4	2.52 ± 0.4	2.93 ± 0.2	3.15 ± 0.3	2.83 ± 0.2	3.54 ± 0.6	2.24 ± 0.2	3.77 ± 0.5
**tZROG**	-	-	-	-	-	-	1.35 ± 0.1	1.25 ± 0.2	1.05 ± 0.1	1.03 ± 0.1	0.7 ± 0.03	1.12 ± 0.2	1.3 ± 0.32	0.7 ± 0.1
**DHZROG**	<lod	<lod	<lod	<lod	<lod	<lod	<lod	<lod	<lod	<lod	<lod	<lod	<lod	<lod
**cZROG**	<lod	0.74 ± 0.2	0.66 ± 0.1	1.29 ± 0.4	8.39 ± 0.7	6.22 ± 1.6	12.3 ± 1.5	3.84 ± 0.5	4.05 ± 0.8	2.58 ± 0.3	13.04 ± 1.4	9.48 ± 0.8	12.5 ± 2.9	28.85 ± 3.9
**tZ9G**	<lod	<lod	<lod	0.12 ± 0.02	0.64 ± 0.2	1.13 ± 0.4	<lod	<lod	0.24 ± 0.1	0.97 ± 0.1	<lod	<lod	0.39 ± 0.1	2.88 ± 0.3
**DHZ9G**	<lod	<lod	<lod	<lod	<lod	<lod	<lod	<lod	<lod	<lod	<lod	<lod	<lod	<lod
**iP9G**	<lod	<lod	<lod	<lod	<lod	<lod	<lod	<lod	<lod	<lod	<lod	<lod	<lod	<lod
**cZ9G**	<lod	<lod	<lod	<lod	<lod	<lod	<lod	<lod	<lod	<lod	0.34 ± 0.02	0.24 ± 0.01	0.34 ± 0.1	0.81 ± 0.1

lod, below the limits of detection; - indicates the values presented in Dhandapani et al. (2017).
